# Hepatitis A and E in rural northern India: clinico-epidemiological and molecular surveillance from a tertiary care hospital

**DOI:** 10.3389/fpubh.2026.1902419

**Published:** 2026-08-17

**Authors:** Shefali Gupta, Priyanka Priyanka, Sana Islahi, Anirudh Mukherjee, Mritunjay Kumar, Sweta Singh, Arti Dwivedi, Niraj Kumar Srivastava, Parul Sinha, Abhijeet Chaudhary, Mukesh Shukla, Namita Mishra, Niraj Kumari

**Affiliations:** 1 Department of Microbiology, All India Institute of Medical Sciences (AIIMS), Raebareli, Uttar Pradesh, India; 2 Department of Internal Medicine, All India Institute of Medical Sciences (AIIMS), Raebareli, Uttar Pradesh, India; 3 Department of Pediatrics, All India Institute of Medical Sciences (AIIMS), Raebareli, Uttar Pradesh, India; 4 Department of General Surgery, All India Institute of Medical Sciences (AIIMS), Raebareli, Uttar Pradesh, India; 5 Department of Obstetrics and Gynaecology, All India Institute of Medical Sciences (AIIMS), Raebareli, Uttar Pradesh, India; 6 Department of Community and Family Medicine, All India Institute of Medical Sciences (AIIMS), Raebareli, Uttar Pradesh, India; 7 Department of Pathology and Laboratory Medicine, All India Institute of Medical Sciences (AIIMS), Raebareli, Uttar Pradesh, India

**Keywords:** acute viral hepatitis, HEV genotype 1a, genotype IIIA, hepatitis A virus, hepatitis E virus, molecular epidemiology, northern India, public health surveillance

## Abstract

**Background:**

HAV and HEV cause substantial preventable morbidity across India, yet contemporary clinico-epidemiological and molecular surveillance from the rural Indo-Gangetic plain is sparse, with no published series from the eastern Uttar Pradesh catchment of AIIMS Raebareli. We addressed this gap through an integrated hospital-based study of acute viral hepatitis (AVH) combining seroprevalence, clinical phenotyping, seasonality and molecular genotyping, with public deposit of all newly generated sequences.

**Methods:**

Serum samples from 554 consecutive patients with clinically suspected AVH at AIIMS Raebareli between January 2023 and October 2024 were screened for anti-HAV and anti-HEV IgM by enzyme-linked immunosorbent assay. Seropositive samples underwent RT-PCR targeting the HAV VP1–VP3 and HEV ORF2 regions, and representative amplicons were Sanger-sequenced. Phylogenetic relationships were reconstructed using maximum likelihood in MEGA 11 with 1,000 bootstrap replicates.

**Results:**

Anti-HAV IgM was detected in 50 patients (9.0%) and anti-HEV IgM in 18 (3.2%); four (0.7%) showed dual seropositivity. The HAV-positive cohort was predominantly pediatric (median 14 years, IQR 7–19), with 44.0% aged 6–15 and 38.0% aged 16–40 years. HEV-positive patients spanned a wider age range (median 13, IQR 4–30). HAV cases peaked pre-monsoon (May–June); HEV occurred year-round. Severe disease was uncommon: one HAV-positive patient developed acute liver failure and died in hospital. RT-PCR confirmed HAV viremia in 11/50 (22.0%) and HEV in 7/18 (38.9%). All sequenced HAV strains were HAV genotype IIIA and HEV strains HEV genotype 1a, with 98–99% nucleotide identity to Indian references.

**Conclusion:**

HAV remains the dominant cause of enterically transmitted AVH in this previously unstudied rural catchment, with disease now concentrated in older children, adolescents and young adults; HEV-1a circulates at lower volume but year-round. Genotypic uniformity (HAV-IIIA, HEV-1a) indicates a stable transmission pattern, and the present sequences (GenBank PV454657–PV454670) extend the molecular record from northern India. Three operational priorities follow: structured HAV immunization of older children and adolescents, a focused pre-monsoon (March–May) WASH outreach window, and routine anti-HEV IgM screening of pregnant women presenting with jaundice.

## 1 Introduction

Hepatitis A virus (HAV) and hepatitis E virus (HEV) account for a sustained burden of acute viral hepatitis (AVH) on the Indian subcontinent. Together these two enterically transmitted viruses account for the bulk of waterborne hepatic illness, driving both endemic transmission and intermittent point-source outbreaks linked to contaminated drinking water and inadequate sanitation (1–3). Although they share a fecal–oral route, their clinical signatures diverge in ways that matter for public-health planning: HAV is classically a self-limiting illness of childhood (1) whereas HEV is associated with severe acute disease in young adults and a disproportionate maternal mortality risk that is unique among the hepatotropic viruses (2, 4–7). On a global scale, HEV is held responsible for roughly 20 million annual infections and according to WHO estimates, approximately 44,000 deaths, with the bulk of that burden falling on low- and middle-income populations in South and Southeast Asia (8).

The epidemiology of HAV in India has shifted measurably over the past two decades. Incremental gains in piped-water access, urbanization and selective vaccination have produced pockets of seronegative adolescents and young adults who present with symptomatic and occasionally fulminant disease on first exposure (9–12). HEV, by contrast, has retained a comparatively stable endemic profile across most of the country, punctuated by waterborne outbreaks during the monsoon and post-monsoon months (3). Hospital-based surveillance from across India reports anti-HAV IgM seropositivity of approximately 7-23% among AVH cases and anti-HEV IgM positivity of 1–11%, with the inter-regional variability driven less by differences in true prevalence than by case-mix, sampling intensity, and the stringency of the AVH case definition applied at admission (13–16).

Molecular surveillance has consistently placed HAV genotype IIIA as the predominant strain circulating across northern, western and eastern India, with low intra-genotype diversity (11, 17, 18). HEV strains in human cases on the subcontinent belong almost exclusively to genotype 1, and within that, subtype 1a (19–21). HEV is a small positive-sense single-stranded RNA virus whose 7.2 kb genome carries three open reading frames; genotype-level variation maps onto the distinct clinical phenotypes seen between the human-restricted, waterborne HEV-1 and HEV-2 lineages and the zoonotic HEV-3 and HEV-4 lineages encountered mainly in industrialized regions (22). Reports of concurrent HAV–HEV infection in the literature are uncommon and most often clinically mild, although individual case series have linked co-infection to a more aggressive hepatic course (23, 24).

Granular surveillance from the rural Indo-Gangetic plain remains scarce despite its epidemiological importance. Uttar Pradesh has the largest population of any Indian state and a high density of communities served by shallow groundwater and incomplete sanitation infrastructure; contemporary hospital-based surveillance from this region is, however, sparse (25), and almost no molecular data exist for the Raebareli catchment. AIIMS Raebareli draws its caseload predominantly from rural communities distributed across several districts of eastern Uttar Pradesh, and is therefore well placed as a sentinel site for the kind of integrated clinical and molecular surveillance that the WHO Global Hepatitis Report 2024 has placed at the centre of the elimination agenda (26).

Against this background, we designed a hospital-based study to address four questions in this catchment: (i) what is the contemporary seroprevalence of HAV and HEV among patients presenting with clinically suspected AVH; (ii) how do the two infections differ in their demographic and clinical presentation; (iii) is there a discernible seasonal pattern; and (iv) which viral genotypes circulate locally. The findings are intended to inform regional vaccination policy, water-and-sanitation prioritization, and the design of molecular surveillance integrated with the National Viral Hepatitis Control Programme.

## 2 Materials and methods

### 2.1 Study design, setting and case definition

We carried out a retrospective, single-centre observational study at the All India Institute of Medical Sciences (AIIMS) in Raebareli, a 600-bed tertiary care teaching hospital whose catchment population is predominantly rural and drawn from across eastern Uttar Pradesh. We included consecutive patients of all ages who presented with clinically suspected acute viral hepatitis (AVH) to the Departments of Internal Medicine, Paediatrics, and the outpatient and emergency services between January 2023 and October 2024, and in whom anti-HAV IgM and anti-HEV IgM serology had been requested as part of routine clinical diagnostic care. De-identified clinical and biochemical data were abstracted from the hospital information system using anonymized alphanumeric codes generated at the point of care, and the residual aliquots of serum used for molecular characterization were retrieved from the Department of Microbiology biorepository. Consecutive enrolment over the full study window was used to minimize selection bias, and the same set of serological assays, RT-PCR primers, cycling conditions and sequencing chemistry was applied throughout the study to ensure procedural homogeneity. Records with missing primary serological results or insufficient residual sample volume were excluded *a priori*. We applied the AVH case definition used in Indian national surveillance (3): an acute illness lasting under 15 days, with any combination of malaise, fever, anorexia, vomiting, nausea, abdominal pain or jaundice, accompanied by serum aminotransferase activity above 2.5 times the upper limit of normal. Patients with serological evidence of current hepatitis B or C infection, known HIV positivity, established chronic liver disease, obstructive jaundice on imaging or insufficient sample volume were excluded.

Within this framework, acute HAV infection was defined by a positive anti-HAV IgM ELISA result, and acute HEV infection by a positive anti-HEV IgM ELISA result, in each case in a patient who met the AVH case definition above. Dual seropositivity for anti-HAV IgM and anti-HEV IgM within a single serum specimen was treated as evidence of co-infection.

### 2.2 Sample handling and clinical data abstraction

Each sample comprised approximately 5 mL of peripheral venous blood drawn under aseptic precautions for diagnostic indication. The serum fraction was obtained by centrifugation at 3,000 rpm for 10 min and aliquoted at the point of care: one aliquot was used immediately for serological testing, while the remaining aliquots were archived at −80 °C in the Department of Microbiology biorepository for subsequent molecular work. De-identified demographic details, presenting symptoms, biochemical investigations (serum bilirubin, AST, ALT, alkaline phosphatase, PT/INR), comorbidities and clinical outcomes were abstracted from the hospital information system using a structured proforma. Where pregnant women with HEV infection were identified in the cohort, maternal and fetal outcomes were retrieved from the corresponding obstetric records held in the hospital information system.

### 2.3 Serological investigations

IgM antibodies against HAV and HEV were detected by IgM-capture enzyme-linked immunosorbent assay (Dia. Pro Diagnostic BioprobesSrl, Milan, Italy), strictly following the manufacturer’s protocol. Concurrent HBV and HCV infection was ruled out for every sample by routine HBsAg screening (MICROLISA; J. Mitra & Co., New Delhi, India) and anti-HCV total-antibody testing (HEPALISA; J. Mitra & Co.). HIV screening was performed using the laboratory’s standard fourth-generation combined antigen/antibody assay. Optical density values were interpreted against the kit-specific cut-off; equivocal results were repeated in duplicate.

### 2.4 Molecular detection of HAV and HEV

Viral RNA was recovered from a 200 μL aliquot of serum with the PureLink Viral RNA/DNA Mini Kit (Invitrogen, Carlsbad, CA, USA) per the manufacturer’s instructions, and cDNA was generated using the QIAGEN OneStep RT-PCR kit (QIAGEN, Hilden, Germany). The synthesized cDNA served as the template for subsequent PCR amplification.

HAV RNA was detected by a conventional RT-PCR assay directed at the VP1–VP3 capsid junction, with an anticipated product of 247 bp. The primer pair was locally designated RBLHA1 (forward, 5′-GTTTTGCTCCTCTTTATCATGCTATG-3′) and RBLHA2 (reverse, 5′-GGAAATGTCTCAGGTACTTTCTTTG-3′). The thermal profile began with initial denaturation at 94 °C for 5 min, followed by 35 cycles of 94 °C / 30 s, 55 °C / 30 s and 72 °C / 45 s, and concluded with a single 5-min extension at 72 °C.

For HEV RNA we used a nested RT-PCR strategy targeting the ORF2 capsid region, with an anticipated final amplicon of 457 bp. The primer set and cycling protocol have been previously applied in Indian HEV molecular surveillance (19). Outer primers were 5′-GGTGGTTTCTGGGGTGACTCC-3′ (forward) and 5′-AGGGGTTGGTTGGATGAAGT-3′ (reverse); inner primers were 5′-GTTGGTGTTTCTGGGGTACCG-3′ (forward) and 5′-AGCCACTCGGCGAAGAACT-3′ (reverse). The first round consisted of an initial denaturation at 94 °C for 5 min, 35 cycles of 94 °C for 30 s, 58 °C for 50 s and 72 °C for 1 min, and a final extension at 72 °C for 5 min. The second round used 30 cycles of 94 °C for 30 s, 64 °C for 25 s and 72 °C for 1 min, with a 5-min terminal extension. All amplifications were performed on a Bio-Rad C1000 Touch thermal cycler (Bio-Rad Laboratories, Hercules, CA, USA). Products were resolved on 2% ethidium bromide-stained agarose gels and observed under UV illumination, with appropriate positive and negative controls included in every run.

### 2.5 Sequencing and phylogenetic analysis

Prior to sequencing, amplicons were cleaned up by exonuclease I–shrimp alkaline phosphatase digestion and ethanol–EDTA precipitation. Bidirectional Sanger sequencing was then performed on an ABI 3500 Genetic Analyzer (Applied Biosystems, Foster City, CA, USA) with BigDye Terminator v3.1 chemistry. Chromatograms were inspected and edited in BioEdit version 7.0.9, and consensus sequences were compiled. Reference sequences spanning all known HAV genotypes (I–VI) and HEV genotypes (1–4) were retrieved from the NCBI GenBank database. Maximum-likelihood phylogenetic reconstruction was carried out in MEGA 11 under the Tamura–Nei substitution model with 1,000 bootstrap replicates. Pairwise nucleotide identity and intra-cluster diversity were calculated using the same software. All nucleotide sequences generated in this study are publicly available from NCBI GenBank: accession numbers PV454657–PV454665 cover the HAV VP1–VP3 amplicons, and PV454666–PV454670 the HEV ORF2 amplicons.

### 2.6 Statistical analysis

Counts and percentages describe categorical variables; for continuous variables, both mean ± standard deviation and median with interquartile range are reported. For each continuous variable we assessed distributional shape using the Shapiro–Wilk test in conjunction with visual histogram inspection; because most variables departed from normality, non-parametric methods were used throughout the analysis. Between-group comparisons (HAV-positive vs. HEV-positive) for categorical variables were performed using Pearson’s chi-squared test; where any expected cell count was less than five, Fisher’s exact test was used in its place. For age, the primary between-group comparison was made on the continuous variable using the Mann–Whitney U test. Age was then grouped into clinically relevant bands to describe the cohort; the *p*-values reported for individual bands are descriptive only and should not be interpreted as formal hypothesis tests. The four continuous biochemical parameters (serum bilirubin, ALT, AST and ALP) were compared between the HAV-positive and HEV-positive groups by the Mann–Whitney U test. All proportions are reported with 95% confidence intervals calculated by the Wilson score method, which provides accurate coverage when sample sizes are small or proportions are close to 0 or 1. All tests were two-sided, with significance set at *p* < 0.05. We used IBM SPSS Statistics (version 26.0; IBM Corp., Armonk, NY, USA) for all statistical analyses.

### 2.7 Ethics statement

Ethics approval for the study was obtained from the Institutional Ethics Committee of the AIIMS, Raebareli on 10 July 2023 (IEC reference 2023-18-IMP-4). The work conforms to the principles of the Declaration of Helsinki and to the 2017 National Ethical Guidelines for Biomedical and Health Research Involving Human Participants issued by the Indian Council of Medical Research. Anti-HAV IgM and anti-HEV IgM serology in patients presenting with acute jaundice formed part of routine clinical diagnostic care at this centre. At admission, the institutional consent form had been signed by every adult patient, and by the parent or legal guardian for any patient under 18 years of age, authorizing the secondary use of de-identified clinical records and residual diagnostic samples for academic research. Participant identifiers were replaced with alphanumeric codes before analysis, and the linkage key was held by the corresponding author alone.

## 3 Results

### 3.1 Cohort characteristics and seroprevalence

Between January 2023 and October 2024, 554 patients with clinically suspected AVH were enrolled and screened for HAV and HEV serology. Anti-HAV IgM was detected in 50 patients (9.0%, Wilson 95% CI 6.9–11.7) and anti-HEV IgM in 18 patients (3.2%, Wilson 95% CI 2.1–5.1). Four patients (0.7%) showed serological evidence of dual infection. Routine screening did not identify any case of HBV or HCV co-infection that required exclusion at the analytical stage.

Sample volume rose progressively across the study window. Of the 554 patients included, 88 (15.9%) were seen during the calendar year 2023 and 465 (83.9%) during January–October 2024; the date of presentation was not recorded for one patient. This pattern reflected the maturation of inter-departmental referral pathways for HAV/HEV testing within the institution rather than under-detection of disease. The seasonality conclusions presented below do not depend on pooling the two calendar years: within the higher-volume 2024 window alone, monthly HAV-positive case counts varied more than fourfold (range 0–13 cases per month) and reproduced the same pre-monsoon peak.

### 3.2 Demographic profile of HAV- and HEV-positive patients

The demographic features of laboratory-confirmed HAV and HEV cases are summarized in Table 1. The HAV-positive group was roughly balanced by sex (male 48.0%, female 52.0%), whereas females accounted for two-thirds (66.7%) of HEV-positive cases; the difference was not statistically significant (*p* = 0.407). The age distribution of the two groups overlapped considerably. The HAV-positive cohort had a median age of 14 years (IQR 7–19), and the HEV-positive cohort a median age of 13 years (IQR 4–30); the Mann–Whitney test gave *p* = 0.867. Stratified by age band, HAV infection was most frequent in children aged 6–15 years (44.0%), with a secondary peak among young adults aged 16–40 years (38.0%). HEV infection was distributed more evenly, with the largest single band being children aged 0–5 years (33.3%) followed by young adults (33.3%). Two HEV-positive women were pregnant at the time of presentation; one delivered a preterm live infant at 32 weeks of gestation, and the other was lost to follow-up after discharge.

**Table 1:** Demographic characteristics of HAV- and HEV-positive patients (AIIMS Raebareli, January 2023 to October 2024).

Variable	HAV positive (*n* = 50)	HEV positive (*n* = 18)	*p*-value*
Sex, *n* (%)			
Male	24 (48.0)	6 (33.3)	0.407
Female	26 (52.0)	12 (66.7)	
Age (years), median (IQR)	14 (7–19)	13 (4–30)	0.867
Age group (years), *n* (%)			
0–5	8 (16.0)	6 (33.3)	0.173
6–15	22 (44.0)	4 (22.2)	0.157
16–40	19 (38.0)	6 (33.3)	0.783
41–60	1 (2.0)	1 (5.6)	0.462

*Fisher’s exact test for categorical variables; Mann–Whitney U test for continuous variables. *α* = 0.05. IQR, interquartile range. No HAV-positive patient and one HEV-positive patient (5.6%) were aged >60 years (*p* = 0.265); not shown above for brevity.

### 3.3 Seasonal distribution

**Figure 1:**
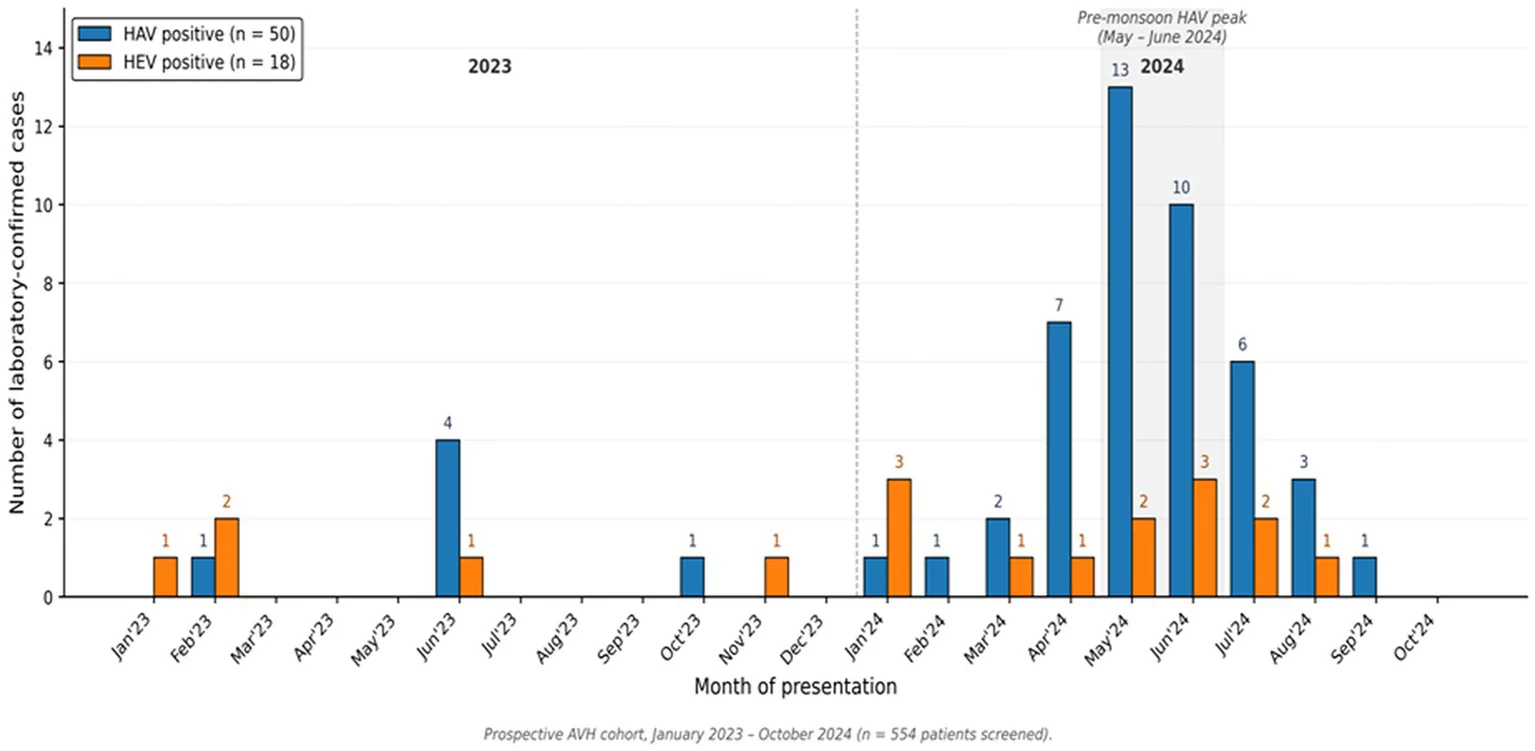
Monthly distribution of laboratory-confirmed HAV and HEV cases at AIIMS Raebareli (January 2023 to October 2024). HAV cases (blue) clustered in the pre-monsoon and early-monsoon months, with 27 of 50 cases (54%) occurring in May and June. HEV cases (orange) showed no comparable seasonal trend and were distributed across most months of the year.

HAV cases showed a clear pre-monsoon and early-monsoon clustering: 27 of the 50 HAV-positive cases (54%) occurred in May and June alone, with a smaller tail extending into July (six cases) and August (three cases). Cases declined sharply between September and February, when the regional burden of waterborne illness is generally lower. HEV cases showed no comparable temporal clustering, with sporadic cases recorded in nearly every month (Figure 1). The seasonality pattern observed for HAV is consistent with run-off contamination of shallow groundwater sources during the early monsoon, when surface water mixing increases.

### 3.4 Clinical manifestations, comorbidities and outcomes

Table 2 summarizes the clinical features and clinical outcomes of HAV-positive and HEV-positive patients. Both groups presented predominantly with classical icteric AVH. Jaundice was the most frequent presenting complaint, recorded in 76.0% of HAV-positive and 55.6% of HEV-positive patients (*p* = 0.134). Fever (58.0% vs. 44.4%), anorexia (58.0% vs. 55.6%), vomiting (38.0% vs. 33.3%) and abdominal pain (34.0% vs. 22.2%) followed in decreasing order. Pruritus, diarrhea, ascites, pedal edema and altered sensorium were each recorded in fewer than 12% of patients in either group, and none of the symptom comparisons reached statistical significance.

**Table 2:** Clinical manifestations, comorbidities and clinical outcomes among HAV- and HEV-positive patients.

Category	Parameter	HAV positive (*n* = 50)	HEV positive (*n* = 18)	*p*-value*
Clinical manifestations	Jaundice	38 (76.0)	10 (55.6)	0.134
	Fever	29 (58.0)	8 (44.4)	0.411
	Loss of appetite	29 (58.0)	10 (55.6)	1.000
	Vomiting	19 (38.0)	6 (33.3)	0.783
	Abdominal pain	17 (34.0)	4 (22.2)	0.553
	Pruritus	3 (6.0)	2 (11.1)	0.602
	Altered sensorium	3 (6.0)	2 (11.1)	0.602
	Ascites	4 (8.0)	0 (0.0)	0.567
	Pedal edema	3 (6.0)	1 (5.6)	1.000
	Diarrhea	4 (8.0)	0 (0.0)	0.567
	Past h/o jaundice	2 (4.0)	1 (5.6)	1.000
Comorbidities	Hepatobiliary disorders	6 (12.0)	3 (16.7)	0.690
	Renal failure	1 (2.0)	0 (0.0)	1.000
	Diabetes mellitus	0 (0.0)	0 (0.0)	—
	Obesity	0 (0.0)	0 (0.0)	—
Severe outcomes	Acute liver failure	1 (2.0)	0 (0.0)	1.000
	Acute-on-chronic liver failure	0 (0.0)	0 (0.0)	—
	Liver transplantation referral	1 (2.0)	0 (0.0)	1.000
	Case fatality	1 (2.0)	0 (0.0)	1.000

Values are number (percentage). *Fisher’s exact test for HAV-positive vs HEV-positive group comparisons. “—” indicates a comparison could not be performed because both groups had a count of zero. No multiple-comparison correction was applied because no comparison reached the uncorrected α = 0.05 threshold; the symptom-level comparisons should therefore be regarded as descriptive.

Comorbid conditions were uncommon in this cohort: associated hepatobiliary disorders (most often acalculous cholecystitis or cholelithiasis on imaging) were noted in 12.0% of HAV-positive and 16.7% of HEV-positive patients, while diabetes mellitus and obesity were not recorded in either subgroup. One HAV-positive adult had concurrent renal failure on admission. Severe hepatic outcomes were rare. One HAV-positive adolescent progressed to acute liver failure (ALF), was referred for liver transplantation, and subsequently died in hospital (case fatality 1/50, 2.0%). During the study period, no HEV-positive patient developed ALF, required transplantation, or died.

### 3.5 Comparative biochemical profile

Liver function test parameters are presented in Table 3. Median total serum bilirubin was higher among HAV-positive patients (1.69 mg/dL, IQR 0.46–5.77) than HEV-positive patients (0.57 mg/dL, IQR 0.38–2.43), although the difference did not reach statistical significance after non-parametric comparison (*p* = 0.116). Median ALT, AST and alkaline phosphatase values were broadly similar between the two seropositive groups (all *p* > 0.10). The wide standard deviations and skewed distributions reflect the small subset of patients with severe hepatocellular injury within each subgroup.

**Table 3:** Comparison of liver function parameters between HAV- and HEV-positive patients.

Parameter	HAV positive (*n* = 50) Mean ± SD [Median (IQR)]	HEV positive (*n* = 18) Mean ± SD [Median (IQR)]	*p*-value*
Total bilirubin (mg/dL)	4.34 ± 6.43 [1.69 (0.46–5.77)]	1.85 ± 2.59 [0.57 (0.38–2.43)]	0.116
ALT (U/L)	264.29 ± 461.94 [50.85 (28.60–262.30)]	143.18 ± 177.53 [46.10 (25.50–196.40)]	0.964
AST (U/L)	299.24 ± 460.88 [51.70 (16.10–412.00)]	163.24 ± 312.91 [41.20 (21.90–88.80)]	0.506
ALP (U/L)	253.78 ± 171.14 [225 (125–336)]	223.88 ± 161.17 [181.5 (86–293)]	0.506

Values are mean ± standard deviation, with median and interquartile range in brackets. *Mann–Whitney U test (HAV-positive vs HEV-positive). ALT, alanine aminotransferase; AST, aspartate aminotransferase; ALP, alkaline phosphatase; IQR, interquartile range; SD, standard deviation.

### 3.6 Molecular detection and phylogenetic findings

**Figure 2:**
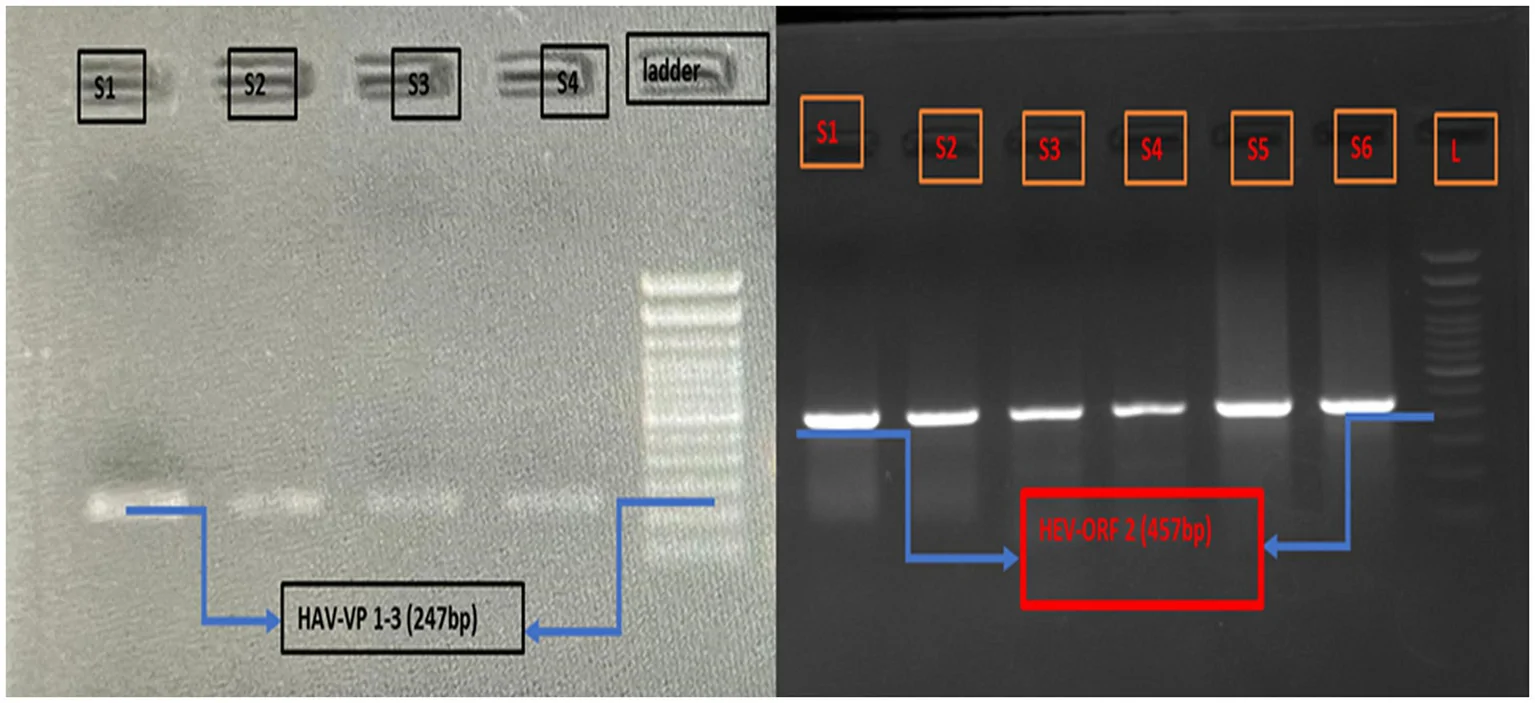
Representative agarose gel electrophoresis showing RT-PCR amplification of HAV and HEV nucleic acids. Left panel: HAV VP1-VP3 capsid junction, 247 bp amplicon; lanes S1-S4, representative patient samples. Right panel: HEV ORF2 capsid region, 457 bp amplicon; lanes S1-S6, representative patient samples. Lane M, 100 bp molecular weight ladder.

All seropositive samples were processed for viral nucleic acid detection. HAV RNA was detected in 11 of 50 anti-HAV IgM-positive samples (22.0%) by conventional RT-PCR targeting the VP1–VP3 region. Nested RT-PCR directed at the ORF2 capsid region recovered HEV RNA from 7 of the 18 IgM-positive samples (38.9%, Figure 2). The lower molecular detection yield among HAV-positive samples is consistent with the typically narrow viremic window in HAV infection, where peak viremia precedes the symptomatic phase by several weeks; samples obtained late in the clinical course frequently fall below detection threshold.

**Figure 3:**
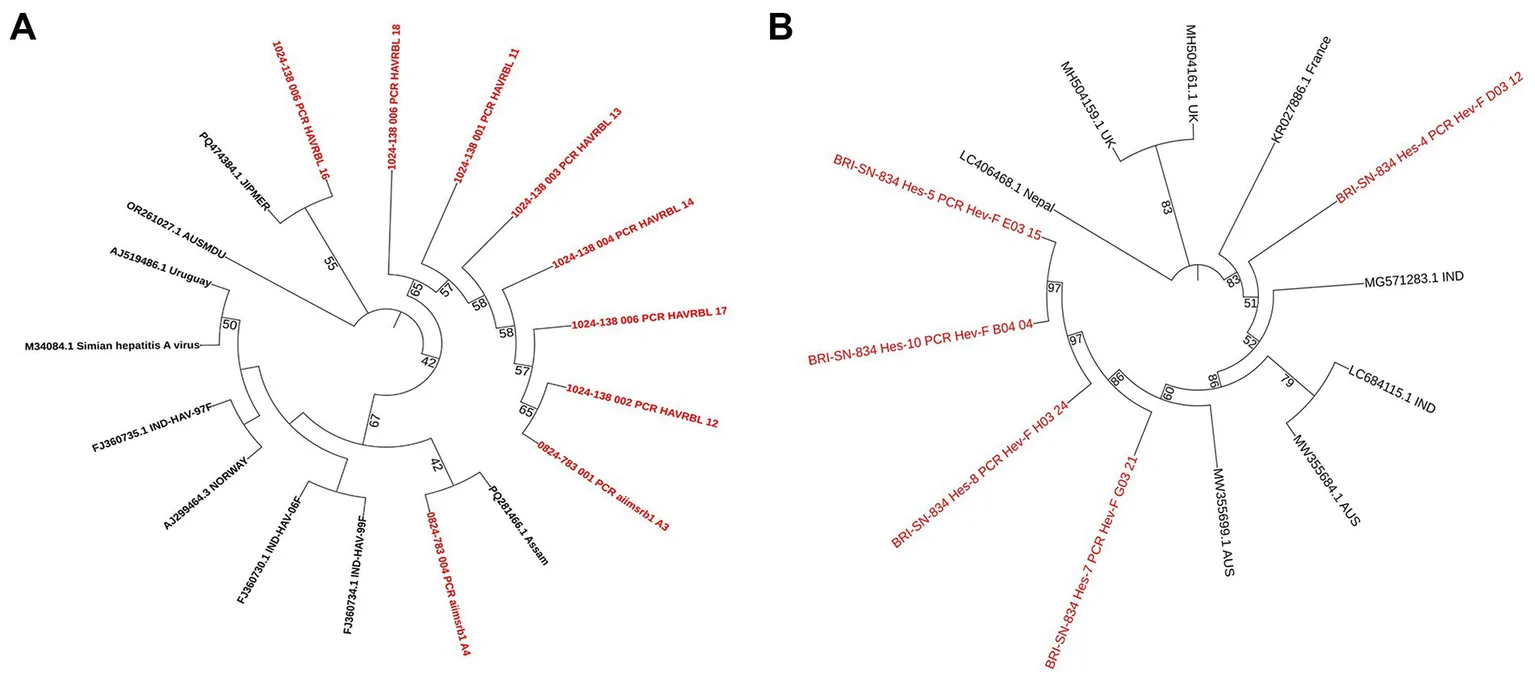
**(A)** Maximum likelihood phylogenetic tree of nine HAV VP1–VP3 sequences obtained from this study (highlighted in red), aligned with reference sequences spanning HAV genotypes I–VI retrieved from NCBI GenBank. The tree was constructed in MEGA 11 using the Tamura-Nei substitution model with 1,000 bootstrap replicates. Bootstrap values >50% are shown at the corresponding nodes. All study isolates clustered within genotype IIIA. **(B)** Maximum likelihood phylogenetic tree of five HEV ORF2 sequences obtained from this study (highlighted in red), aligned with reference sequences covering HEV genotypes 1–4. The tree was constructed in MEGA 11 using the Tamura-Nei substitution model with 1,000 bootstrap replicates. All study isolates clustered within HEV genotype 1a alongside Indian, Nepalese, and Australian reference strains.

Sanger sequencing was successfully completed on nine HAV and five HEV amplicons of sufficient quality. Maximum-likelihood phylogenetic reconstruction (Figure 3A) placed all nine sequenced HAV isolates within genotype IIIA, the predominant subgenotype circulating across India. Pairwise nucleotide identity with the Indian reference strains FJ360730.1 (IND-HAV-06F) and PQ261466.1 (Assam) was 98–99%, with mean intra-cluster nucleotide diversity of 2–4% across the VP1–VP3 region. Bootstrap support for the IIIA clade ranged from 42 to 67%. All five sequenced HEV strains clustered within HEV genotype 1a (Figure 3B) alongside Indian (MG571283.1), Nepalese (LC406468.1) and Australian (MW355691.1) reference sequences. Bootstrap support for the 1a clade ranged from 79 to 97%. Across both HAV and HEV alignments, no evidence of mixed-genotype infection or recombination was detected.

## 4 Discussion

HAV and HEV are responsible for the majority of waterborne viral hepatitis worldwide primarily transmitted by contaminated food and water. As granular surveillance from the rural Indo-Gangetic plain is limited, this hospital-based study aims to explore the contemporary seroprevalence of HAV and HEV among clinically suspected AVH patients, the difference in their demographic and clinical presentation, seasonal pattern; and the locally circulating viral genotypes.

To our knowledge, this is the first integrated clinico-epidemiological and molecular characterization of acute hepatitis A and E from the rural Raebareli catchment of eastern Uttar Pradesh; the accompanying HAV-IIIA and HEV-1a sequences are also the first from this catchment to be entered into NCBI GenBank. Earlier molecular surveillance from urban tertiary centres in Lucknow (18, 19) and Chandigarh (17) has documented the dominance of HAV-IIIA and HEV-1a in northern India; our data extended this picture to a contemporary (2023–2024) rural catchment with a distinct sanitation and exposure profile. Four observations frame the analysis that follows. Hepatitis A remains the principal etiology of enterically transmitted AVH at this tertiary centre. The disease has shifted into older children and adolescents, with a secondary cluster in young adults. Hepatitis E circulates at lower frequency but year-round and across a wider age range, and remains an important risk in pregnancy. The HAV-IIIA and HEV-1a genotypes detected here match those documented elsewhere in India (11, 19), with no evidence of imported lineages or recombination. This study therefore fills a known geographic gap in molecular surveillance of enterically transmitted hepatitis in the rural Indo-Gangetic plain, compares the clinical and biochemical phenotypes of HAV- and HEV-positive patients within a single catchment and study window, and identifies a discrete pre-monsoon HAV peak that can serve as a seasonal focus for water-and-sanitation outreach.

The 9.0% HAV and 3.2% HEV seroprevalence figures fall within the range reported by other Indian tertiary centres over the past decade. Palewar and colleagues recorded 6.7% HAV and 8.5% HEV in western India (13); Sharma and colleagues 11.2 and 1.8% in Himachal Pradesh (14); Jain and colleagues 22.9 and 9.8% in central India (16); and a one-year study from King George’s Medical University, Lucknow reported 7.93% HAV (18). The variability across these estimates is more informative than any single value. It reflects differences in the case-mix admitted, the stringency with which the AVH case definition is applied, the season of sampling, and, almost certainly, the local sanitation gradient (25). Inferences about national prevalence drawn from any single tertiary cohort therefore overstate what hospital-based data can reveal; together, these series show that endemic transmission remains pervasive across India, with regional intensity shaped by catchment-specific exposures.

The age structure of the HAV cohort is the most striking epidemiological finding. A generation ago, almost all symptomatic HAV in India would have been expected to occur in the under-fives, with adult disease the rare exception. We instead found 44.0% of cases concentrated in 6–15-year-olds and 38.0% in young adults aged 16–40 years, with only 16.0% in the under-fives. Grover and colleagues at the Institute of Liver and Biliary Sciences in Delhi documented the same trend on a much larger scale, identifying a steady rise in adolescent and adult HAV admissions across more than 14,000 patients over a nine-year period (10). This pattern is consistent with the demographic consequences of India’s middle-income transition: gradual improvements in piped water and sanitation push first exposure into adolescence and young adulthood (9), enlarging an immunologically naïve cohort in whom symptomatic disease, sometimes complicated by ALF, replaces the subclinical childhood seroconversion of earlier decades. Healthcare workers in Uttar Pradesh remain almost universally seropositive for IgG anti-HAV by adulthood (12), confirming that exposure is still ubiquitous; what has shifted is the timing of infection, not the eventual probability of being infected.

The age distribution of HEV cases was distinctly bimodal, and the median masks this. Although the overall median age of 13 years may look inconsistent with the classical young-adult peak of HEV-1 illness, it reflects two separate age concentrations rather than a general shift toward younger patients: 33.3% of HEV-positive cases were aged 0–5 years and a further 33.3% were aged 16–40 years. The young-adult concentration matches the established peak incidence of HEV-1 in endemic regions (2). The paediatric weighting of the median is driven by the unexpected under-five cluster, which sits awkwardly with the textbook view that pediatric HEV is largely subclinical. The most likely explanation is referral bias: pediatric admissions to a tertiary centre with biochemical hepatitis preferentially capture the symptomatic minority of pediatric HEV exposures, and the population-level frequency cannot be inferred from these data alone. Two HEV-positive women in the cohort were pregnant; one delivered preterm. The sample is too small for inferential statistics, but the literature linking HEV-1 to maternal–fetal morbidity and excess maternal mortality (6, 27) is consistent enough that systematic anti-HEV IgM screening of any pregnant woman presenting with jaundice in this region would, we suggest, be worth its modest cost. Chronic HEV is well documented in immunocompromised hosts in industrialized settings (8), but our cohort displayed no such cases, consistent with the genotype 1 phenotype that dominates the subcontinent.

Seasonality differed sharply between the two viruses. HAV cases concentrated in May and June, with 27 of the 50 HAV-positive cases (54%) presenting in those two months across the 22-month study window, a pre-monsoon and early-monsoon clustering consistent with surface-water and shallow-groundwater contamination during runoff (3, 15, 25). HEV cases, by contrast, were distributed across nearly every month of the study window, suggesting continuous low-level transmission, multiple unrelated exposures, or a combination of imported and locally acquired infection. The contrast has direct operational consequences. A pre-monsoon WASH intervention (chlorination of shallow groundwater, point-of-use filtration, food-handler hygiene) applied for a six-week window in March to May would cover the bulk of HAV transmission in this catchment. HEV control needs a different approach: sustained interventions across the year, with particular attention to water sources serving high-risk populations such as pregnant women.

Biochemically, the HAV-positive group showed higher median serum bilirubin (1.69 mg/dL) than the HEV-positive group (0.57 mg/dL), a difference also observed in a larger European comparison, in which total bilirubin was 1.4 times higher in HAV than in HEV (28). The difference was not statistically significant by Mann–Whitney (*p* = 0.116), and we read it as a directional rather than statistical finding: the small HEV subgroup limits power, and the wide variance reflects a few patients with severe hepatocellular injury whose extreme values pulled the means upward in both groups. Aminotransferase distributions overlapped substantially, and no biochemical parameter reliably distinguished HAV from HEV at presentation. In practice, biochemistry should not be a substitute for serology in the differential diagnosis of acute jaundice in this catchment.

Severe hepatic outcomes (ALF, transplant evaluation and in-hospital death) were rare. One HAV-positive adolescent progressed to ALF and died; no HEV-positive patient experienced any of these endpoints. The 2.0% case-fatality rate for HAV falls broadly within the range described across Indian hospital-based series, in which reported mortality varies widely with case-mix and referral pattern (3, 10, 15) and contrasts with specialist liver-unit cohorts that capture a more severely selected case-mix (10). For a general tertiary care catchment that admits the unselected mix of pediatric, obstetric and adult presentations, this low-severity profile is expected: most HAV in this age range is self-limiting, ALF is the rare exception, and case fatality concentrates in the few patients who develop encephalopathy. Clinically, HAV in the rural Indo-Gangetic plain is dominated by manageable acute illness rather than fulminant disease. The absolute number of ALF events, however, applied across the population that AIIMS Raebareli serves, remains substantial.

Molecular characterization produced a consistent and reassuring picture. All sequenced HAV strains belonged to genotype IIIA, the same genotype identified by Lole and colleagues across multiple Indian states (11), by Singh et al. from northwestern India (17), and by Prakash et al. from Lucknow (18). All sequenced HEV strains belonged to subtype 1a, matching the predominant subtype reported across the Indian subcontinent and adjacent South Asian countries (19, 20, 22). A whole-genome study from Bangladesh similarly demonstrated that endemic and outbreak HEV-1a strains form a tight regional cluster with limited diversity, suggesting sustained transmission of a small set of related lineages across the subcontinent (29). Our data fits that pattern. Pairwise nucleotide identity of 98–99% with Indian reference sequences and low intra-cluster diversity within each genotype point to stable transmission, with neither imported lineages nor recombination events apparent in the dataset. Diagnostically, licensed HAV vaccines should retain their effectiveness in this catchment for the foreseeable future (30); the diagnostic primers used in this study should likewise remain suitable, and any deviation from this pattern in future surveillance would itself be informative.

The proportion of seropositive samples in which we recovered viral RNA (22.0% for HAV and 38.9% for HEV) falls within the published Indian range. Singh et al. reported 47% HAV-RNA recovery from anti-HAV IgM-positive samples in Chandigarh (17); Prakash et al. reported 46% in Lucknow (18); and Bhatnagar et al. reported 32% HEV-RNA recovery from anti-HEV IgM-positive samples (19). Two factors probably explain the lower RT-PCR positivity in IgM-seropositive samples. First, the narrow viremic window of both viruses caps what any hospital-based molecular surveillance system can detect; this is particularly true for HAV, where peak viral load occurs during the late incubation period and falls steeply after symptom onset, so samples obtained at first clinical presentation are frequently drawn after peak viremia has subsided. Second, anti-HAV and anti-HEV IgM antibodies persist in serum for weeks to months after viremia clears, so a share of IgM-positive samples are expected to be RT-PCR-negative on biological grounds; this does not affect the seroprevalence estimates or the phylogenetic conclusions drawn from the RT-PCR-positive samples. Improving RT-PCR yield in future surveillance would require sampling earlier in the disease course, ideally during the prodromal phase, an objective that outbreak-investigation protocols can achieve more readily than routine hospital-based diagnostic testing.

Four patients (0.7%) carried serological evidence of dual HAV-HEV infection, a proportion closely comparable to the 0.5-0.6% reported from western India and Himachal Pradesh (13, 14), though considerably higher rates have been described in some Indian series. None of the co-infected patients in our cohort experienced severe outcomes. Although case reports describe accelerated hepatic decompensation in dual infection (23), a sample of four cannot establish or refute clinical excess risk. It does, however, confirm that co-infection is uncommon in this catchment but not absent, and that recognizing the pattern requires concurrent rather than sequential serological testing.

Three priorities emerge from this work for the regional public-health response. First, the documented age shift in HAV incidence supports a structured immunization program covering older children and young adolescents, who now constitute the bulk of symptomatic disease and an immunologically vulnerable group in this catchment (12, 30). Although HAV vaccine is not yet part of India’s Universal Immunization Programme, the live attenuated formulation is licensed in India and recommended for routine pediatric administration from 18 months of age (30); the cost-effectiveness case for broader public-sector inclusion strengthens with each additional report of adult-onset symptomatic disease. Second, the pre-monsoon HAV peak supports time-limited WASH outreach concentrated in the March–May window (chlorination of shallow groundwater, point-of-use filtration and food-handler hygiene), which would not only attenuate HAV but also reduce the broader burden of waterborne illness (1, 26). Third, the relative invisibility of HEV in routine surveillance, set against its disproportionate impact on pregnant women, supports systematic anti-HEV IgM screening of any pregnant woman presenting with jaundice or biochemical hepatitis (6, 27), together with sustained investment in molecular surveillance integrated with the National Viral Hepatitis Control Programme. These recommendations are based on data from a single rural catchment and should be treated as preliminary rather than definitive; confirmation in multi-centre studies and formal policy assessment would be needed before wider implementation.

Several limitations apply. The single-centre design relies on de-identified clinical records and archived diagnostic samples, with sample selection driven by clinician request rather than systematic screening; the findings therefore reflect what was clinically suspected and tested for, and are not directly extrapolable to community incidence or to the burden of subclinical infection. The same narrow viremic window may also have led us to under-count HAV–HEV co-infection. Bootstrap support for the HAV-IIIA clade was modest (42–67%), attributable to the short amplicon used and the limited variation within a single subgenotype; pairwise nucleotide identity of 98–99% with Indian reference sequences nevertheless placed all study isolates within IIIA without ambiguity. The HEV-positive subgroup (n = 18) was small, including only two pregnant patients. We did not follow patients beyond the index clinical encounter, and cannot therefore describe infection progression, resolution, chronicity in immunocompromised sub-groups, or long-term outcomes; prospective studies with structured follow-up would give a more complete picture. Finally, the absence of full-genome sequencing limits the resolution of any minor subgenotype shifts or recombination events; a multi-site sentinel network with next-generation sequencing across Uttar Pradesh would address this gap and strengthen the evidence base for national hepatitis elimination planning (26, 29).

## 5 Conclusion

This study confirms that HAV remains the principal driver of enterically transmitted acute viral hepatitis at a rural tertiary centre in northern India and documents a marked epidemiological shift of HAV into older children, adolescents and young adults rather than the under-fives. HEV-1a circulates at lower volume but year-round and across a wider age range, and remains the more important pathogen for pregnant women presenting with jaundice. The genotypic uniformity observed (HAV-IIIA, HEV-1a, both with high sequence identity to Indian reference strains and no evidence of imported lineages or recombination) points to a stable transmission pattern that is amenable to targeted intervention. Three priorities follow directly from these data: structured HAV immunization of older children and adolescents, pre-monsoon (March–May) WASH outreach, and routine anti-HEV IgM screening of pregnant women presenting with jaundice. All would benefit from sustained molecular surveillance integrated with the National Viral Hepatitis Control Programme.

## Data Availability

The datasets presented in this study can be found in online repositories. The names of the repository/repositories and accession number(s) can be found in the article/supplementary material.
